# Extensive Macular Atrophy with Pseudodrusen Imaged with OCT Angiography

**DOI:** 10.1155/2018/8213097

**Published:** 2018-10-23

**Authors:** Jaclyn L. Kovach

**Affiliations:** Bascom Palmer Eye Institute, Miller School of Medicine, University of Miami, 3880 Tamiami Trail N, Naples, FL 34103, USA

## Abstract

This report describes the first case of extensive macular atrophy with pseudodrusen (EMAP) imaged with optical coherence tomography angiography (OCTA). A 58-year-old Caucasian man presented with decreased central vision in both eyes. Fundus examination showed large areas of macular atrophy centered on the fovea surrounded by diffuse reticular pseudodrusen. Spectral domain OCT (SDOCT) revealed outer retinal and choriocapillaris atrophy. OCTA demonstrated marked absence of choriocapillaris flow. Extensive macular atrophy with pseudodrusen is a rare clinical entity and a new extreme phenotype of macular degenerations that could shed more light on the role of pseudodrusen and choriocapillaris compromise in the pathogenesis of AMD.

## 1. Introduction

Extensive macular atrophy with pseudodrusen (EMAP) is a rare clinical entity that was first described by Hamel et al. in 18 patients with bilateral well-delineated chorioretinal atrophy extending to the temporal arcades without sparing the fovea and widespread pseudodrusen throughout the posterior pole and peripheral retina. Earlier onset, more rapid progression of atrophy, and severe visual loss were noted in contrast to age-related macular degeneration (AMD) [1]. Choroidal neovascularization is a possible complication that responds to anti-VEGF therapy [2].

Optical coherence tomography angiography (OCTA) is a relatively new, fast, noninvasive imaging modality that analyzes high-speed OCT images, measures changing reflectance, and reconstructs high-resolution blood flow maps of the retina, allowing en face imaging of the retinal capillary plexuses and choroidal vasculature.

The author describes the first case of EMAP imaged with optical coherence tomography angiography (OCTA).

## 2. Case Report

**Figure 1:**
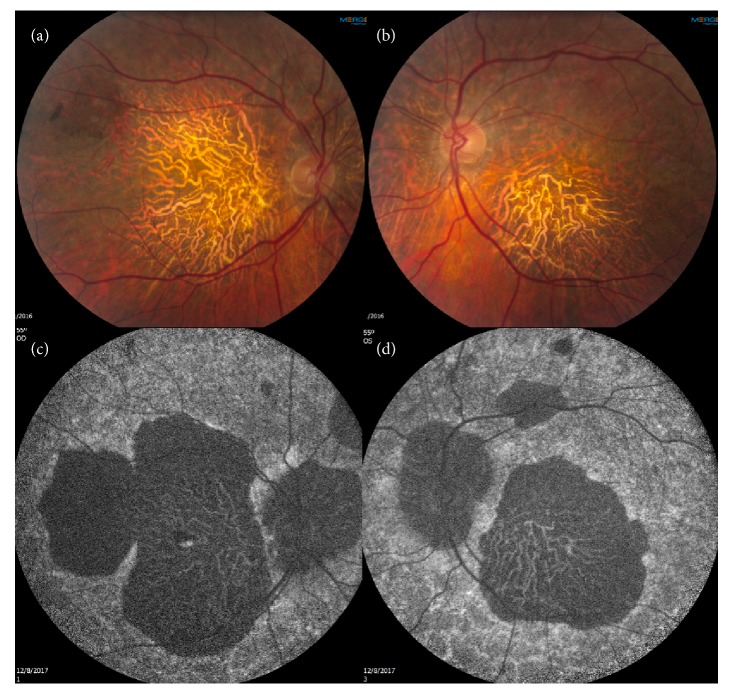
(a) and (b) Color fundus photo demonstrating macular atrophy and surrounding pseudodrusen OU. (c) and (d) FAF reveals hypoautofluorescence in areas of atrophy OU with a central island in the right macula.

**Figure 2:**
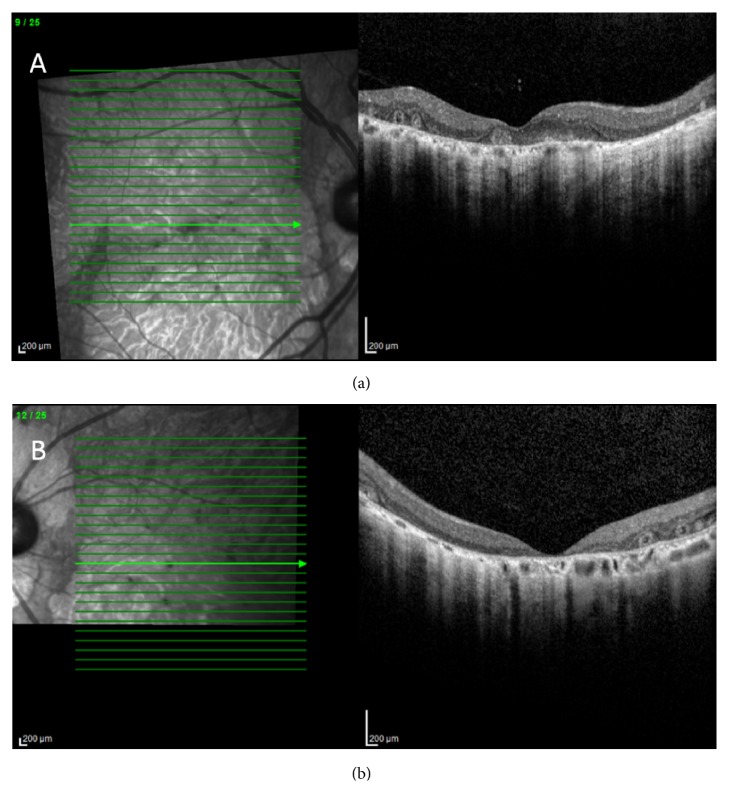
(a) and (b) SDOCT shows outer retinal and choriocapillaris atrophy with outer retinal tubulations OU.

**Figure 3:**
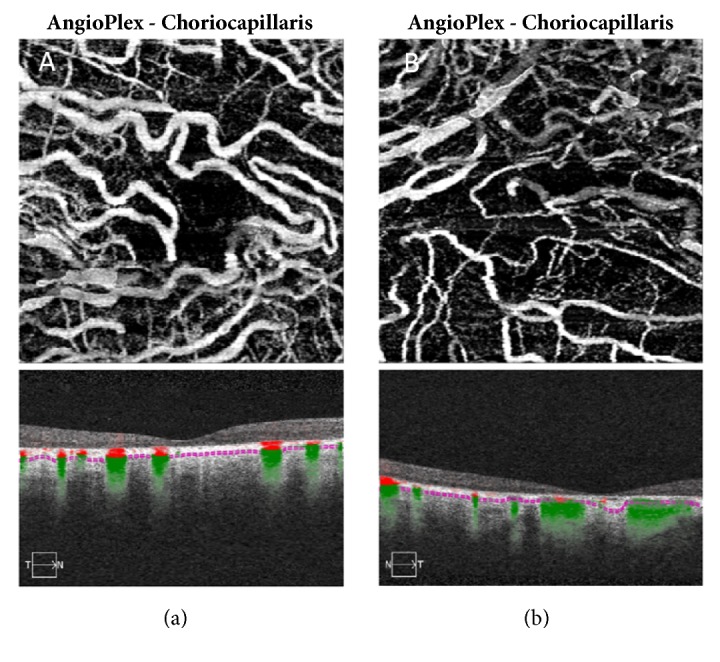
(a) and (b) OCTA reveals a marked decrease in choriocapillaris flow with large choroidal vessel flow present in the right (a) and left (b) eyes.

A 58-year-old Caucasian male presented with progressively decreasing central vision in both eyes over the past five years. He also complained of mild night blindness. There was no significant medical history and no family history of retinal disease. Best-corrected visual acuity was 20/60 in the right eye and 20/200 in the left eye with mild nuclear sclerotic cataracts. Fundoscopic examination revealed large areas of macular atrophy centered on the fovea surrounded by reticular pseudodrusen. Peripapillary atrophy was also present along with scattered areas of peripheral pavingstone degeneration OU (Figures 1(a) and 1(b)). There was no intraocular inflammation and a recent electroretinogram (ERG) was within normal limits. Fundus autofluorescence (FAF) showed well-defined areas of atrophy OU and sparing of the central fovea in the right eye (Figures 1(c) and 1(d)). Spectral domain OCT (SDOCT) (Heidelberg Spectralis OCT, Heidelberg, Germany) revealed outer retinal and choriocapillaris atrophy and reticular pseudodrusen (Figures 2(a) and 2(b)). OCTA (Angioplex, Carl Zeiss Meditec, Dublin, CA) demonstrated marked absence of choriocapillaris flow (Figures 3(a) and 3(b)). The retinal vasculature was mildly attenuated on OCTA as well. These vascular changes were not readily apparent on fluorescein angiography. Genetic testing for the A3243G and C1QTNF5 mutations was negative.

## 3. Discussion

Extensive macular atrophy with pseudodrusen (EMAP) is a retinal dystrophy that affects patients in their sixth decade and is defined by bilateral symmetric widespread macular atrophy centered on the fovea surrounded by diffuse macular and midperipheral pseudodrusen. Pavingstone lesions can also be found peripherally [3]. An association between EMAP and a family history of AMD and glaucoma, a female predominance, and a systemic inflammatory profile has been documented [1]. Consumption of a Mediterranean diet could be protective. A cohort of 65 patients with EMAP underwent genetic analysis for common and rare AMD risk alleles but a significant association was not found [4]. Fundus autofluorescence is helpful in identifying the extent of macular atrophy and OCTA is able to detect the marked reduction in choriocapillaris flow that would be expected given the inner choroidal thinning present on SDOCT.

Extensive macular atrophy with pseudodrusen may be a new extreme phenotype of macular degenerations that could shed more light on the role of pseudodrusen and choriocapillaris compromise in the pathogenesis and progression of AMD. An association between pseudodrusen and decreased choroidal thickness has been documented [5] as has a correlation between pseudodrusen and geographic atrophy (GA) in AMD and AMD progression [6]. A reduction in CH50 plasma levels and increased C3 in patients with EMAP could indicate a more severe complement pathway dysfunction compared to AMD and play a role in increased pseudodrusen formation and atrophy progression [1]. Given the subretinal origin of pseudodrusen, it is difficult to know the location of the inciting event in pathogenesis for either condition: internal to the retinal pigment epithelium or in the choroid. A multimodal imaging approach to evaluating EMAP and other degenerative macular diseases, which includes OCTA, has the potential to foster greater insight into the pathogenesis of these potentially devastating conditions.
